# Cancer immunotherapy trials: leading a paradigm shift in drug development

**DOI:** 10.1186/s40425-016-0146-9

**Published:** 2016-07-19

**Authors:** Leisha A. Emens, Lisa H. Butterfield, F. Stephen Hodi, Francesco M. Marincola, Howard L. Kaufman

**Affiliations:** Department of Oncology, Sidney Kimmel Comprehensive Cancer Center, Johns Hopkins University School of Medicine, 1650 Orleans Street, Room 409, Baltimore, MD 21231-1000 USA; Departments of Medicine, Surgery, and Immunology, Hillman Cancer Center, University of Pittsburgh, Pittsburgh, PA USA; Dana Farber Cancer Institute, Boston, MA USA; Sidra Medical and Research Center, Doha, Qatar; Division of Surgical Oncology, Rutgers Cancer Institute of New Jersey, New Brunswick, NJ USA

## Background

Cancer immunotherapy has a long history in the clinic. William Coley was an orthopedic surgeon in the late 1800’s who noted marked tumor regression in a patient with severe erysipelas. Based on this observation, he hypothesized that stimulating the immune system could effectively treat cancer. Thus, over the next decades he gave various bacterial products, including live bacteria, to patients with advanced cancers [1]. Although toxicity was often substantial, he and his colleagues noted tumor regressions in some patients, particularly those with bone and soft tissue sarcomas. His work was groundbreaking in strategy and method, but eventually fell out of favor due to lack of scientific control and the advent of radiotherapy and chemotherapy for cancer treatment. Despite this, he was a pioneering physician-scientist. His work is notable because his hypothesis that the immune system can be harnessed to treat cancer is the bedrock of the striking success of cancer immunotherapy today.

In contrast to his relatively unsystematic approach, drug development has historically followed a carefully delineated pathway of sequential trials that systematically generate data characterizing the safety profile and clinical activity of a therapeutic agent in increasing numbers of patients [2]. These trials begin with early Phase 1 studies in small numbers of patients that evaluate the toxicities, pharmacokinetics (PK) and pharmacodynamics (PD) of a novel agent, and define a dose and schedule for further evaluation. Exploratory Phase 2 studies enroll greater numbers of patients to further characterize the side effect profile and estimate the anti-tumor activity of the therapy, typically in a given tumor type. Randomized Phase 3 clinical trials then enroll larger numbers of patients with a specific type of cancer, comparing the new therapy to placebo and/or the current standard of care to confirm the safety and clinical activity of the new intervention. This drug development system is designed to support the marketing approval of more active and/or less toxic cancer therapies for patients based on Phase 3 clinical trial data in large numbers of patients. Phase 4 post-marketing studies aim to generate additional safety and clinical efficacy data for marketed products.

The need for rapid and efficient cancer drug development has never been greater. The sequencing of cancer genomes has identified multiple rearranged or mutated targets that are druggable, ushering in the era of precision medicine. Concomitantly, advances in our knowledge of the molecular and cellular basis of the antitumor immune response has driven the development of multiple single-agent cancer immunotherapies. These agents target nonmalignant immune cells, and have often shown striking clinical activity across multiple tumor histologies. Importantly, the activities of targeted therapies and immunotherapies frequently intersect, with molecularly targeted therapies often promoting the antitumor immune response. Moreover, the activity of both precision medicines targeting genomic aberrations in tumor cells and immunotherapies targeting the antitumor immune response is maximized by the development of companion diagnostics that identify patients most likely to benefit from them. This may require the development of a companion diagnostic through a parallel regulatory pathway, or may be done simultaneously in registration clinical trials. Given the array of available targets, drugs, and biomarkers of response and resistance to therapy it is clear that traditional approaches to drug development are unable to support the speed and level of sophistication required for the development of effective cancer therapies today.

New trial designs more effectively and efficiently tie the biologic activity of new agents to clinical activity and are more appropriate in today’s landscape than the traditional phased approach based on associations between toxicity and clinical activity [3]. Platform trials evaluate multiple therapies in a given indication alone or in combination, and may evolve over time to test new agents and drop drugs with limited activity or excessive toxicity. Indication finding trials evaluate therapies across histologies or disease subtypes within a given histology to define the most appropriate indications for further development. Adaptive trials are conducted as unblinded trials that flex to incoming data in real time based on prospectively defined decision rules. Importantly, early first-in-human Phase 1 clinical trials increasingly include expansion cohorts designed to test hypotheses distinct from the historical objectives of traditional Phase 1 clinical studies [2, 4]. These cohorts may be designed to explore more sophisticated PK and PD parameters, alternative drug dose and schedule schemas, improved product formulation or administration strategies, predictive biomarkers of therapeutic response and resistance, and assessment of clinical activity. Most recently, these trials have begun to add large expansion cohorts of 100 patients or more [2]. The total sample size of these clinical trials may grow to exceed well over 1000 patients. The addition of new expansion cohorts to first-in-human trials to test emerging clinical hypotheses in real time can clearly expedite the development of new cancer therapies, delivering better treatments to patients much faster.

Strategies for meeting the challenges and mitigating the possible risks of this strategy must be identified and developed with the input from all stakeholders. This is particularly true for combination cancer immunotherapies that integrate immuno-oncology agents with standard chemotherapy or radiation, molecularly targeted therapies, or other immuno-oncology agents. These combination immunotherapy strategies are rapidly entering the clinic. Importantly, they have the potential for therapeutic synergy, but also a significant risk of enhanced and unexpected toxicities. The optimal integration of modern translational, clinical, and regulatory science is essential for delivering new single agent and combination cancer immunotherapies to patients as safely and efficiently as possible.

## Case studies of cancer immunotherapy trials

The clinical success of immune checkpoint antagonists targeting the PD-1 pathway in multiple tumor types provides clear evidence that harnessing the immune system can lead to durable tumor regressions in cancer patients. As a result, enthusiasm for cancer immunotherapy has never been higher, and complex trials of single agent and combination cancer immunotherapies are entering the clinic at a dizzying pace. George Santayana once said, “Those who fail to learn from history are doomed to repeat it.” This may be true for the prior failures and risks of novel cancer immunotherapies. We also argue that those who fail to learn from history will not develop better ways of moving forward. We should apply lessons from our successes to create the most effective pathway for developing cancer immunotherapies. Here we present several case studies in cancer immunotherapy drug development to highlight important lessons from the past. These lessons illustrate  innovative, rapid clinical development pathways for novel immunotherapy agents with clinical benefit highlight the need for appropriate cautions to detect and manage potential adverse events early in the drug development process.

### Unique pathways to drug approval

#### The PD-1 antagonist pembrolizumab

The clinical development of pembrolizumab is an example of a modern, seamless drug development strategy that facilitates the rapid testing and approval of a novel, highly promising agent under the umbrella of a single clinical trial [2, 3]. The first-in-human study began in 2011 to define the recommended phase 2 dose for advanced solid tumors. A high level of clinical activity observed in the patients initially enrolled led to an increase in the sample size for patients with melanoma in order to evaluate dose characteristics, overall response rates, and disease control rates for this disease. Similarly, significant clinical activity in non-small cell lung cancer (NSCLC) prompted the addition of a cohort of patients with NSCLC to estimate the overall response rate in this second disease indication. Additional cohorts of patients with other types of cancer were added, and ultimately over 1200 patients were treated on this open-label Phase 1b trial [5]. One cohort enrolled 173 patients with unresectable or metastatic melanoma previously treated with ipilimumab or BRAF/MEK inhibitors as appropriate, randomizing them to 2 mg/kg or 10 mg/kg of pembrolizumab every 3 weeks [6]. Initially patients were randomized at a 2:1 ratio, and then the protocol was adjusted to achieve a 1:1 randomization. The primary endpoint for these cohorts was the overall response rate by RECIST. Peak and trough blood samples were obtained for pharmacokinetic analysis. The overall response rate and safety profiles were similar for both doses tested, though the peak drug exposure was higher for the 10 mg/kg dose cohort. The clinical efficacy observed in this cohort was sufficient to support accelerated approval of pembrolizumab at 2 mg/kg every 3 weeks for unresectable or metastatic melanoma in September 2014, only 3 years after the trial began. Subsequently data from the NSCLC cohort [7] led to accelerated approval of pembrolizumab for metastatic PD-L1+ NSCLC with a companion diagnostic, the PD-L1 IHC 22C3 pharmDx test, in October 2015. This unusually rapid path to marketing approval for pembrolizumab in two disease indications was possible due to the striking clinical responses observed and the durability of clinical benefit, and included breakthrough therapy designation, the thoughtful use of expansion cohorts, the development of a companion diagnostic assay for at least one tumor type, and an accelerated approval strategy developed in close communication with the FDA.

#### The PD-1 antagonist nivolumab

Similar to pembrolizumab, the clinical development path of nivolumab, another PD-1 inhibitor, was also shortened. Ultimately, accelerated approval in unresectable or advanced melanoma was granted in December 2014 based on clinical efficacy data derived from a subset of 120 patients enrolled on a randomized Phase 3 clinical trial that screened 631 patients at 90 sites across the world [3].

#### The oncolytic virus talimogene laherparepvec (T-VEC)

T-VEC, a genetically-modified oncolytic herpesvirus encoding granulocyte-macrophage colony-stimulating factor (GM-CSF), is the first oncolytic virus to demonstrate clinical benefit in a prospective, randomized phase III clinical trial [8]. T-VEC has a novel mechanism of action that includes both the direct lysis of tumor cells and the induction of systemic host anti-tumor immunity [9]. The clinical development of T-VEC in metastatic melanoma required considerable thought to define the eligible patient population, which required accessible tumor for direct injection of virus, attention to pre-existing herpes virus serology, and the measurement of viral shedding to detect household transmission. The pivotal phase III trial also required a control arm and a clinical endpoint appropriate to a novel class of immunotherapy agent, as earlier phase studies of T-VEC had demonstrated disease progression before regression in some patients [10]. In the phase III trial, 436 patients were enrolled using a 2:1 randomization to T-VEC or recombinant GM-CSF (selected as the control in this study). In this trial the 2:1 randomization was utilized for efficiency of clinical development in a compromise between the use of historical controls and randomized concurrent controls, where the concurrent randomized controls help to confirm that historical controls are an appropriate comparison group and the level of imbalance in the randomization reflects the level of confidence in historical control data [11]. The trial was completed within a 2-year period. Patients were required to have accessible but unresectable melanoma, and were allowed to continue treatment despite clinically insignificant disease progression. The primary endpoint was durable response rate, defined as an objective complete or partial response by modified WHO criteria beginning within 12 months of starting study treatment and lasting at least 6 months from the time of onset. This trial demonstrated a significant improvement in durable response rate, with improvements in objective response rate, progression-free survival and quality of life. Subset analyses revealed an especially strong effect on response rate and overall survival for patients with stage III or IVM1a disease [7]. Based on the durable response rate and quality of life improvements (psychological distress) associated with regression of cutaneous and soft tissue disease, the FDA approved T-VEC for patients with advanced melanoma in October 2015. The drug was also approved by the European Medicines Agency in December 2015 for the treatment of patients with advanced stage III and IVM1a melanoma only, based on the subset analyses. This trial illustrates the use of a novel clinical endpoint that incorporates both response and duration elements to capture the known delayed kinetics of clinical responses often observed with immunotherapy agents.

### Unique toxicities and complications

#### The CD28 superagonist TGN1412

The clinical application of agents that target regulatory molecules within the immunologic synapse began well before the current era of immune checkpoint modulation targeting the CTLA-4 and PD-1 pathways (among others). TeGenero developed a first-in-class humanized superagonist monoclonal antibody specific for CD28, TGN1412 [12, 13]. CD28 is a cell surface receptor that engages with its ligands CD80 and CD86 on professional antigen presenting cells to stimulate T cell responses during antigen recognition. TGN1412 induces very strong CD28 signaling that requires only tonic antigen-specific T cell receptor (TCR) signaling. Thus, the majority of the signal for T cell activation is delivered through CD28 rather than through the TCR. TGN1412 entered the clinic on March 13, 2006, when six healthy volunteers received an intravenous injection of the CD28 superagonist at about the same time. They all developed a life-threatening cytokine-release syndrome (CRS), and were admitted to the intensive care unit within 12–16 h after dosing; all six survived. This unexpected and life-threatening toxicity immediately led all involved to ask why preclinical data did not predict this life-threatening toxicity, and it took 6 years to dissect the science.

In preclinical models, CD28 superagonists result in polyclonal T cell activation that is rapidly counter-regulated by the expansion and activation of regulatory T cells (Treg) to prevent over-exuberant immune activation. Several peculiarities of the preclinical data lend insight into what happened. First, in the healthy volunteers, tissue-resident CD4+ effector memory T cells (T_EM_) were the source of cytokines (IL-2, TNFα, and IFN-γ) resulting in CRS. Because the accumulation of T_EM_ is driven by repeated exposure to infectious agents, it does not occur in rodents housed in clean rooms. Thus, the relative T_EM_/Treg ratio in laboratory rodents and in humans differs, and favors immune hyperstimulation in patients. Moreover, low dose superagonist CD28 only expands Tregs, whereas doses at the level used in the trial expands both T_EM_ and Tregs. Thus, the dose selected for study compounded the risks associated with the biological difference between rodents and humans. Second, TGN1412 had been given to cynomolgus macaques at 500-fold higher doses than to the human volunteers without adverse effect. Human and macaque CD28 has the same sequence and binds TGN1412 with the same affinity. However, macaque CD4+ T cells, but not human CD4+ T cells, lose CD28 expression as they differentiate into T_EM_ cells. This difference was overlooked during primate testing. Finally, treating human peripheral blood mononuclear cells with TGN1412 did not induce cytokine release or proliferation. Subsequent investigations showed that when PBMC were “pre-conditioned” at high density to facilitate cell-cell contact mimicking tissue residence, the sub-threshold, tonic level of T cell activation required for the activity of TGN1412 that T cells lose during trafficking through the circulation was restored. Pre-culturing PBMC under high density conditions suggests that if TGN1412 had been given at the minimum anticipated biological effect level (MABEL), the first dose for the trial would have been 200-fold lower. This trial taught us that “the absence of evidence” is not the same as “evidence of absence” for drug-related toxicity [13], arguing that receptor occupancy assays and in vitro surrogate response markers—dosing based on the minimum biologic effect level (MABEL)—may be superior to the no observed adverse effect level (NOAEL)-based dosing used in this trial.

#### CAR-T cell therapy

Chimeric antigen receptor T (CAR-T) cell therapy is a highly active cancer immunotherapy that yields dramatic tumor regressions. CAR-T cells are T cells engineered to express an artificial antigen receptor derived from the variable region of an antigen-specific antibody and linked to signaling components of the T cell receptor and co-stimulatory molecules for T cell activation. Like TGN1412, CAR-T cell therapy can result in massive T cell expansion in vivo, leading to CRS [14, 15]. CAR-T cell therapy is also associated with macrophage activation syndrome (MAS) or hemophagocytic lymphohistiocytosis (HLH), and a previously unrecognized form of focal neurologic toxicity. These are not antigen-specific toxicities, but are associated with high circulating levels of several cytokines, including interleukin-6 and interferon-γ. Interleukin-6 has emerged as the central mediator of severe CRS-associated toxicity, and therapy for CRS includes tocilizumab and corticosteroids along with aggressive supportive care. Despite knowledge of the pathophysiology of this syndrome, its management remains a challenge. In 2014, clinical trials of CAR-T cells were temporarily put on hold at Memorial Sloan Kettering Cancer Center due to several infusion-related patient deaths, prompting modifications to the protocol [16]. This included reducing the dose of CAR-T cells in patients with large tumor burdens, as the degree of tumor load appeared to correlate with the severity of CRS. The protocol was also modified to exclude patients with a history of cardiac problems, and decreased the time period between the end of induction chemotherapy and the start of CAR-T cell therapy by 50%. It also prompted the development of a consensus statement for the management of CRS from leaders in the field [17]. In addition to CRS and associated syndromes, T cell therapy may be associated with other unexpected toxicities [18]. An affinity-enhanced TCR for MAGE-3 under development for melanoma and myeloma caused cardiogenic shock and death in the first two patients who received it due to cross-reactivity to an unrelated peptide, titin, on striated muscle [19].

#### The listeria-based vaccine axalimogene filolisbac

A *Listeria monocytogenes*-based bacterial vaccine targeting human papilloma virus (HPV) has been tested in patients. In one clinical trial, a patient with cervical cancer was treated on study, with the last dose given in early 2013. In July 2015 the patient was hospitalized, and tests discovered the engineered *Listeria* bacteria in her bloodstream. The patient died in August 2015. The death was attributed to disease progression, but the FDA put four clinical trials testing axalimogene filolisbac on clinical hold in October 2015. The presence of axalimogene filolisbac in the bloodstream of this patient was ultimately attributed to colonization of the biofilm overlying an implanted medical device, seeding the device for re-emergence of the agent at a later date. Based on evaluation of the event, the protocol inclusion/exclusion criteria were modified to exclude patients with indwelling medical devices that are a high risk for biofilm colonization and cannot be easily removed. Study procedures were modified to require the strict use of a dedicated indwelling line for administration of the agent, a 6-month course of antibiotics after the last dose of axalimogene filolsibac was implemented, and additional patient surveillance and longer term monitoring measures were put in place. The clinical hold was lifted in December 2015.

## Challenges and opportunities

The accelerated approvals described above illustrate how the timeline of drug development can be compressed using innovative adaptive clinical trial designs that enroll large numbers of patients in early trials coupled effectively with ongoing consultation with regulatory authorities. In large part due to these triumphs of drug development, there are now dozens of clinical trials testing novel immunotherapies alone and in combination with other therapies for the first time. This may include the first in human testing of two novel immunotherapy agents with the aim of testing them in combination under the umbrella of a single protocol. The trials often grow to include sites that span the globe with investigators who may have relatively limited experience in cancer immunotherapy. The vignettes above also highlight the importance of early detection of potential safety issues, the need to implement effective communication strategies amongst the study team, and the importance of efficiently implementing adverse event management guidelines into clinical trials as new data is accumulated. The progress we have made and the number of immunotherapy agents entering the pipeline thus poses challenges in how to prioritize them, study them most safely and effectively, and develop the most rational immunotherapy combinations for clinical testing. Here, we discuss these challenges and propose some strategies for meeting them.

### Clinical endpoints

Advances in tumor immunotherapy have been dramatic over the last five years. We now understand that the kinetics of immune-mediated anti-tumor activity may be delayed compared to that of cytotoxic chemotherapy or targeted therapy. Immunotherapy mediates tumor regression indirectly through activation of immune responses and/or inhibition of suppressive immune elements. This may result in delayed tumor regression, with some patients even experiencing the progression of existing disease or the appearance of new lesions prior to eventual disease regression. Furthermore, many immunotherapy agents do not impact progression-free survival, but are associated with significant improvements in overall survival [20]. Standard metrics for tumor response assessment thus may not capture the true clinical benefit of cancer immunotherapies, and the selection of clinical endpoints may require re-thinking when immunotherapy agents are being used as part of a cancer treatment regimen.

The use of alternative clinical endpoints for immunotherapy has already been widely discussed. First, the atypical response patterns of cancer immunotherapy led to the development of the immune-related response criteria (irRECIST), which captures disease in bidimensional measurements and allows for continued treatment in the face of clinically insignificant disease progression [21]. Immune-related RECIST (irRECIST) was subsequently simplified to modified RECIST criteria (mRECIST) capture disease in unidimensional measurements while still allowing for the unique response patterns of cancer immunotherapies with apparent disease progression by standard criteria that is clinically insignificant (delayed response and/or pseudoprogression) [22]. A recent report from the pembrolizumab Phase 1b study demonstrated a significant rate of atypical responses, where standard RECIST criteria appeared to underestimate the benefit of pembrolizumab in about 15% of patients [23]. Thus, accounting for the atypical response patterns of cancer immunotherapy might prevent the cessation of treatment in patients who will go on to benefit from cancer immunotherapy. Second, the durable response rate is a novel clinical endpoint already successfully incorporated into the registration strategy for T-VEC. It captures response rates over a pre-specified time frame based on anticipated time to disease progression, which may differ across various cancer types. Importantly, the strong association between stable disease and improved overall survival increasingly reported for cancer immunotherapy further supports disease control rate as a highly clinically relevant endpoint. Disease control rate may be a particularly appropriate endpoint in early studies of novel single agent and combination cancer immunotherapies [24]. A caveat to the use of disease control rate as an endpoint for clinical trials is that it may be confounded by indolent disease biology: in the absence of a randomized control arm, it could overestimate clinical benefit. One way to mitigate this risk is the use of an imbalanced randomization such as that used in the pivotal trial leading to the approval of T-VEC. Third, the unusually high rate of complete responses seen in some recent combination immunotherapy trials suggests that complete response could emerge as an appropriate clinical endpoint for selected cancer immunotherapy strategies [25]. Finally, composite endpoints that capture both the depth and duration of response may be most appropriate for some cancer immunotherapies. Efforts to develop novel endpoints appropriate to the mechanism of action of the particular immunotherapy strategy being tested are ongoing.

### Patient selection/eligibility

The selection of patients for cancer immunotherapy trials is key to their success. Four considerations are relevant to cancer immunotherapy trials today. First, in the past most trials enrolled patients who were immunotherapy naïve. As these trials became more widespread, newer trials started to exclude patients who had received prior cancer immunotherapies. Given the growth of the field and recent marketing approvals, this is likely no longer feasible as a matter of course, and patients previously treated with one agent may respond to re-challenge with the same agent, or to another drug. Thus, the exclusion of patients who have received prior immunotherapy should be based on a strong scientific rationale, or immunotherapy naïve and experienced patients could be studied in separate study cohorts. Second, after initial reports that PD-L1 expression within the tumor microenvironment had potential as a predictive biomarker, many sponsors defined eligibility for trials of PD-1 or PD-L1 antagonists based on expression of PD-L1 within the tumor using a pre-defined assay. Selection for intratumoral PD-L1 expression does appear to enrich for responses, and likely helped to ferret out clinical signals in early studies. However, PD-L1 expression is not a perfect predictive biomarker. Four Phase 3 clinical trials in melanoma, NSCLC, and RCC have evaluated PD-L1 expression as a predictive biomarker of response to therapy with PD-1 pathway antagonists [25–28]. In these trials, higher PD-L1 expression is associated with more clinical responses and longer survival, but significant numbers of PD-L1 negative patients also benefit from blockade of PD-1 signaling. Thus, a significant challenge to the use of PD-L1 expression as a predictive biomarker of response is that it is not a binary variable. The level of PD-L1 expression for a given patient may be more prognostic than predictive, with higher levels associated with better clinical outcomes. Assay differences, expression of PD-L1 by both tumor cells and infiltrating immune cells, geographic variability of PD-L1 expression within the tumor microenvironment, cellular localization of PD-L1 (membranous versus cytoplasmic), and the dynamic nature of the immune response lend further complexity to the development of immunologic biomarkers that reliably predict response to immunotherapy. It is likely that a composite signature may be most informative. Finally, the relationship between PD-L1 expression and clinical benefit is likely to vary with tumor type. PD-L1 did not predict clinical benefit with nivolumab in renal cell carcinoma [28], but did in melanoma and selected subtypes of lung cancer but not others [25–27]. Third, incorporating high quality biomarker programs designed to identify predictive biomarkers of primary or secondary resistance into these trials can help to prioritize the most rational immunotherapy combinations for clinical development [29]. Fourth, given the high level of excitement about immune checkpoint blockade, patients increasingly are asking to be treated off-label with approved agents, or under a single patient IND for investigational agents. Many of these patients may present with PD-L1 testing results on their tumor done as part of a trial, using one of the two diagnostic tests that have now been approved by the FDA, or even another assay. The lack of standardization for PD-L1 testing and the dynamic nature of PD-L1 expression may create substantial uncertainty in determining the relevance of the result at a given point in time for a particular patient. Given both the opportunities at hand and data that support a higher quality of care for patients on trial, clinical trial participation should be strongly encouraged for potentially eligible patients to ensure the field continues to move forward at a rapid pace and we bring the best drugs to the most appropriate patients.

### Trial modifications

The rapid growth and sheer size of these trials present challenges to communication, ensuring that investigators, sponsors, contract research organizations, investigational review boards and regulatory authorities remain up-to-date on the current data and modifications to the protocol, that patients are fully informed, and that patient safety and data quality are maximized. As trials are modified, the rationale, supporting data, and hypotheses to be tested in the amendment must be clearly defined. This could include evaluating additional tumor types for signals of clinical activity, modified drug doses or schedules, and/or predictive biomarkers of response or resistance (among other questions). The sample size justification and statistical analysis plan should be described, and early stopping rules for futility and/or toxicity clearly defined. Study participants should be kept informed of new developments in the study that warrant major changes to the study design through updated informed consent documents. Scientific review committees, institutional review boards, and the appropriate regulatory authorities should be notified of major changes to the study design and sample size/patient cohorts being evaluated. A plan for patient follow up should also be clearly delineated to collect late safety and efficacy signals.

### Trial communications

In cancer immunotherapy trials, unexpected (and expected) toxicities can quickly emerge, requiring an informed, early, and nimble response from the investigator for maximal patient safety. These events may also provide a limited window for evaluating patient samples that can elucidate the pathophysiology of an unexpected or rare event. Effective real-time communication between the study investigators and the sponsor will facilitate dissecting the science in the patient to inform the development of the most effective therapy for a specific adverse event (such as the use of tocilizumab for CRS). Additionally, the atypical clinical response patterns that may be associated with cancer immunotherapy can be tricky to manage for investigators, and difficult for patients to understand. For investigators with less experience in immunotherapy, having defined management algorithms and an infrastructure to fall back on to guide both toxicity management and the management of atypical clinical responses is critical. A defined communications strategy for cascading information about important adverse events (expected or unexpected) as well as atypical response patterns should be implemented early in the study. Regularly scheduled “state of the study” calls have proven very useful to make sure all stakeholders are optimally informed. The communications between the sponsor and investigators across multiple sites that occur on these calls are highly valuable. Monthly trial newsletters and toxicity management hotlines are other strategies that have been used to inform and support study investigators faced with serious immune-related toxicities new to them.

### Trial oversight

An excellent, well-defined communications plan including all trial stakeholders is the first step to rigorous trial oversight. A steering committee that includes study investigators should also be considered to provide critical advice and insights from individuals with expertise in the field and “on-the-ground” experience with the study, particularly for new agents or novel immunotherapy combinations. Once a study incorporates advanced design features (randomization, for example) and/or matures sufficiently that it could support a registration for marketing approval, it may be appropriate to consider an oversight committee that includes external experts with subject matter expertise who are not directly involved in the study. These individuals can provide an objective voice in monitoring the data and the evolution of the study design to ensure that the safety and clinical activity signals seen are considered appropriately across all disease cohorts, and the clinical science is maintained at the highest level of quality. These strategies are similar to historic data safety monitoring boards but demand more frequent updates and interaction among all stakeholders.

## Conclusions

Recent advances in cancer immunotherapy clearly illustrate virtually unprecedented long-term clinical benefit in some advanced cancer patients, and the potential for cure seems tangible. In lock step with transforming patient care, we have also already begun to revolutionize the drug development enterprise. Given the sheer number of investigational agents and potential combination cancer immunotherapies before us, the opportunity to make a major impact on the world’s cancer burden and on patient’s lives has never been greater. This is an exciting time in the field, and it is critical to expedite the development of new cancer immunotherapies. It is also imperative to remain vigilant in order to detect safety signals early, and incorporate adverse event management procedures rapidly to prevent avoidable toxicity. We should continue to re-structure and adapt the system to fit the opportunities before us, working as a team across the drug development enterprise to safely deliver transformative cancer immunotherapies to the patients and families that need them. We should learn from past experiences and recent successes, and communicate emerging clinical trial data often and clearly among investigators, local review boards and federal regulators. These strategies will help to ensure that we maintain the highest level of ethical and scientific rigor as the clinical development of cancer immunotherapy evolves and we continue to innovate to eradicate cancer.
